# Identification of hub genes and therapeutic drugs in esophageal squamous cell carcinoma based on integrated bioinformatics strategy

**DOI:** 10.1186/s12935-019-0854-6

**Published:** 2019-05-22

**Authors:** Wanli Yang, Xinhui Zhao, Yu Han, Lili Duan, Xin Lu, Xiaoqian Wang, Yujie Zhang, Wei Zhou, Jinqiang Liu, Hongwei Zhang, Qingchuan Zhao, Liu Hong, Daiming Fan

**Affiliations:** 1 State Key Laboratory of Cancer Biology, National Clinical Research Center for Digestive Diseases, and Xijing Hospital of Digestive Diseases, Fourth Military Medical University, Xi’an, China; 2 Department of Otolaryngology, Xijing Hospital, Fourth Military Medical University, Xi’an, China; 3 The School of Life Science and Technology, Xi’an Jiaotong University, Xi’an, China

**Keywords:** Esophageal squamous cell carcinoma, Bioinformatics, Hub genes, Cell cycle, Differentially expressed genes, Drug

## Abstract

**Background:**

Esophageal squamous cell carcinoma (ESCC) is one of leading malignant cancers of gastrointestinal tract worldwide. Until now, the involved mechanisms during the development of ESCC are largely unknown. This study aims to explore the driven-genes and biological pathways in ESCC.

**Methods:**

mRNA expression datasets of GSE29001, GSE20347, GSE100942, and GSE38129, containing 63 pairs of ESCC and non-tumor tissues data, were integrated and deeply analyzed. The bioinformatics approaches include identification of differentially expressed genes (DEGs) and hub genes, gene ontology (GO) terms analysis and biological pathway enrichment analysis, construction and analysis of protein–protein interaction (PPI) network, and miRNA–gene network construction. Subsequently, GEPIA2 database and qPCR assay were utilized to validate the expression of hub genes. DGIdb database was performed to search the candidate drugs for ESCC.

**Results:**

Finally, 120 upregulated and 26 downregulated DEGs were identified. The functional enrichment of DEGs in ESCC were mainly correlated with cell cycle, DNA replication, deleted in colorectal cancer (DCC) mediated attractive signaling pathway, and Netrin-1 signaling pathway. The PPI network was constructed using STRING software with 146 nodes and 2392 edges. The most significant three modules in PPI were filtered and analyzed. Totally ten genes were selected and considered as the hub genes and nuclear division cycle 80 (NDC80) was closely related to the survival of ESCC patients. DGIdb database predicted 33 small molecules as the possible drugs for treating ESCC.

**Conclusions:**

In summary, the data may provide new insights into ESCC pathogenesis and treatments. The candidate drugs may improve the efficiency of personalized therapy in future.

**Electronic supplementary material:**

The online version of this article (10.1186/s12935-019-0854-6) contains supplementary material, which is available to authorized users.

## Background

Esophageal cancer (EC) ranks seventh in terms of incidence and sixth in cancer deaths worldwide, responsible for about 572,000 new cases and 509,000 deaths last year [1]. Although we have made great progress on the early diagnosis and novel therapy, EC still is one of challengeable diseases in Eastern Asian [1]. Generally, EC includes two most common histologic subtypes: esophageal squamous cell carcinoma (ESCC) and esophageal adenocarcinoma (EAC) [2]. ESCC comprises over 90% of all EC cases [1]. And risk factors, such as smoking and hot drinks, are closely related to the initiation of ESCC [1, 2]. However, the underlying mechanisms of ESCC are not well understood. And due to the lack of specific biomarkers, most ESCC patients are diagnosed at a late stage, leading to particularly poor outcomes of patients [3]. Even worse, some of ESCC patients suffer from tumor recurrence due to the chemotherapy resistance [3]. Therefore, it is of paramount importance to find novel biomarkers and effective targets for ESCC patients.

Recently, gene profile and gene chip have been extensively applied in the field of scientific researches [4, 5]. Gene expression analysis based on these methods can quickly detect the differentially expressed genes (DEGs) that may have a strong influence on cancer progression [6]. However, most of the gene chip or gene profile data have been only deposited in public databases. And re-analyzing these data can be an efficient way to provide the new insights into further studies. So far, many studies have used gene chip or gene profile to identify key genes for ESCC, and numerous DEGs have been detected [7]. Nevertheless, the results may be inconsistent and variable because of the existence of tumor heterogeneity. To date, few reliable biomarkers and therapeutic targets have been identified for ESCC [8]. Thus, it’s urgent to discover new markers and therapeutic targets for ESCC patients.

Many chemotherapeutic drugs have shown activity against ESCC, including docetaxel [9–11], cisplatin [10, 11], fluorouracil [9–11], and nedaplatin [9]. Moreover, the combinations of these agents are also recommended because of the existence of chemotherapy resistance. A recent study found that concurrent chemoradiotherapy (CCRT) with 5-fluorouracil plus cisplatin were more effective and less toxic than CCRT with the docetaxel plus cisplatin as the first-line treatment for ESCC patients [11]. However, the progression-free survival and overall survival (OS) of ESCC patients remained short, highlighting the importance of developing some molecular drugs.

In the study, four mRNA expression profiles were downloaded (GSE29001 [12], GSE20347 [13], GSE100942 [14], and GSE38129 [15]) from GEO database, from which there are 63 pairs of ESCC and non-tumor tissues data available. Integrated analyses included identifying DEGs using the GEO2R tool, overlapping four datasets using a Venn diagram tool, GO terms analysis, biological pathway enrichment analysis, PPI construction, hub genes identification and verification, miRNA–hub genes network construction, and exploration of the candidate small molecular drugs for ESCC.

## Materials and methods

### Data collection

ESCC and adjacent normal tissue gene expression profiles of GSE20347 [13], GSE29001 [12], GSE100942 [14], and GSE38129 [15] were downloaded from GEO (http://www.ncbi.nlm.nih.gov/geo/) database [16]. The microarray data of GSE29001 was based on GPL571 Platforms (Affymetrix Human Genome U133A 2.0 Array) and included 12 pairs of ESCC and non-tumor tissues (Submission date: May 02, 2011). The GSE20347 data was based on GPL571 Platforms (Affymetrix Human Genome U133A 2.0 Array) and included 17 ESCC tissues and 17 normal tissues (Submission date: Feb 16, 2010). The GSE100942 data was based on GPL570 Platforms (Affymetrix Human Genome U133 Plus 2.0 Array) and included 4 ESCC tissues and 4 non-tumor tissues (Submission date: Jul 07, 2017). The GSE38129 data was based on GPL571 Platforms (Affymetrix Human Genome U133A 2.0 Array) and included 30 pairs of ESCC and non-tumor tissues (Submission date: May 22, 2012). The above datasets met the following criteria: (1) they used tissue samples from human ESCC tissues and paired adjacent or non-tumor tissues; (2) each dataset involved more than eight samples.

### DEGs identification

GEO2R (https://www.ncbi.nlm.nih.gov/geo/geo2r/) was used to pick out the DEGs in ESCC tissues and adjacent non-tumor tissues [17]. p < 0.05 and |logFC| > 1 were set as the cut-off criterion to select DEGs for every dataset microarray respectively [7, 17]. Finally, the overlapping DEGs among the four datasets was identified by Venn diagram tool (http://bioinfogp.cnb.csic.es/tools/venny/).

### Cell culture, RNA extraction and quantitative PCR (qPCR)

Human ESCC cell line EC109 and human esophageal squamous epithelial cell line Het-1A were cultured in RPMI-1640 medium (Gibco) with 10% fetal bovine serum (Gibco) at 37 °C in a humidified atmosphere with 5% CO_2_. Total RNA was extracted from cells using the E.Z.N.A.™ Total RNA Kit I (OMEGA). PrimeScript™ RT Master Mix (Perfect Real Time) was used for RNA reverse transcription. SYBR Premix Ex Taq (TaKaRa) was employed to conduct qPCR assay. PCR primers were designed and synthesized by TaKaRa (Additional file 1: Table S1). The experiments were performed in three times. GAPDH was used as the internal control.

### GO and biological pathway enrichment analysis

GO terms analysis of selected DEGs were performed using the DAVID database (https://david.ncifcrf.gov/; version: 6.8) [18]. We submitted the DEGs, including 120 upregulated genes and 26 downregulated genes, into DAVID with p < 0.05 as the cut-off criterion. The GO results of significant terms for cellular component (CC), biological process (BP), and molecular function (MF) were ranked by p-value and exhibited as bar charts. The FunRich tool (version: 3.0) was mainly used for analyzing the functional enrichment and interaction networks of genes and proteins [19]. In this study, the FunRich was used to analyze the biological pathways of DEGs. Finally, the top 10 biological pathways of upregulated genes and downregulated genes were presented as bar charts, respectively. p-value < 0.05 was considered as statistically significant.

### PPI network construction and analysis

PPI networks are the networks of protein complexes formed as the results of biochemical or electrostatic forces [20]. PPI network is crucial for molecular processes, and abnormal PPI is the basis of many diseases, including tumors [21]. In this study, the Search Tool for the Retrieval of Interacting Genes (STRING) database (https://string-db.org/cgi/input.pl; version: 11.0) [20], Cytoscape software (version: 3.6.1) [22], and FunRich were utilized to construct PPI networks. Cytoscape and FunRich tool were applied to present the PPI networks with the cut-off criterion as confidence score ≥ 0.4, maximum number of interactors = 0. The Molecular Complex Detection (MCODE) plug-in of Cytoscape tool was employed to visualize the significant gene modules in ESCC with degree cutoff = 2, node score cutoff = 0.2, k-core = 2, and max. depth = 100. The criteria for selecting the top 3 significant modules were set as follows: MCODE scores ≥ 4 and number of nodes ≥ 4 [23]. FunRich tool was performed to do the functional enrichment for each module. 10 hub genes with high degree of connectivity were selected and mapped into PPI based on STRING following confidence score ≥ 0.4, maximum number of interactors ≤ 5. Furthermore, STRING was used to perform the co-expression analysis of hub genes.

### Validation of the hub genes

The GEPIA2 (http://gepia2.cancer-pku.cn/#index) is an online database for analyzing gene expression profiles of 9736 tumors and 8587 normal samples from the Cancer Genome Atlas (TCGA) and the genotype-tissue expression (GTEx) projects [24]. Thus, we can validate the expression levels and genes correlations of hub genes in ESCC tissues and normal tissues. The cBio Cancer Genomics Portal (http://www.cbioportal.org/; version: 2.2.0) is an open access tool which provides analysis, visualization, and downloads of cancer genomics datasets of many types of tumors [25]. Complex cancer genomics profiles are accessible from the cBioPortal tool, thus enabling us to compare the genetic alterations of the selected ten hub genes in ESCC.

### miRNA–hub gene network

The targeted miRNAs of hub genes were predicted by four established miRNA target prediction databases [miRanda, PITA, PicTar, and TargetScan (version: 3.1)]. The miRNAs predicted by at least two programs were selected as the targeted miRNAs of hub genes. A co-expression network based on correlation analysis of hub genes and miRNAs associated with cancer was constructed by Cytoscape software. In the network, a green circular node represented the miRNA and a red circular node represented the hub gene, their interaction was represented by an arrow. The numbers of arrows in the networks indicated the contribution of one miRNA to the surrounding genes, and the higher the degree, the more central the hub gene was within the network.

### Drug-hub gene interaction

The 10 hub genes were also served as the promising targets for searching drugs through the DGIdb (http://dgidb.genome.wustl.edu/; version: 3.0.2—sha1 ec916b2) [26]. This database contains drug-gene interaction data from 30 disparate sources including ChEMBL, DrugBank, Ensembl, NCBI Entrez, PharmGKB, PubChem, clinical trial databases, and literature in NCBI PubMed. The results of this process were arranged so that each entry was a specific drug-gene interaction associated with its source link [27]. Drugs supported by more than one databases or PubMed references were selected as the potential drugs. The final list only involved the drugs that have been approved by the Food and Drug Administration (FDA). The identified target network was visualized using STITCH (http://stitch.embl.de/; version: 5.0), a program similar to STRING which also incorporated drug-gene relationships [27, 28].

## Results

### Identification of DEGs

**Fig. 1:**
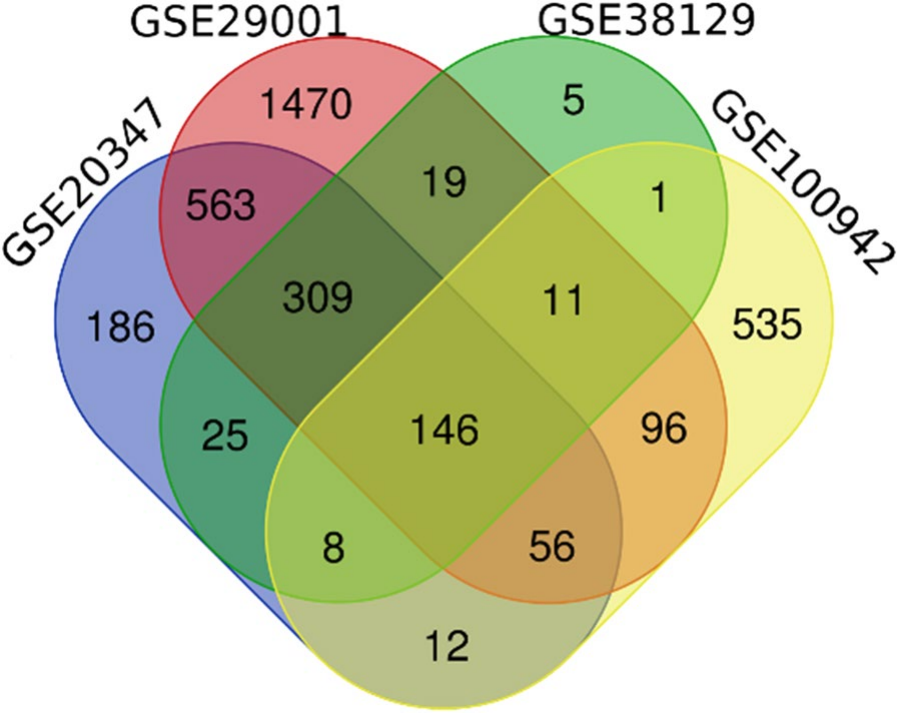
Identification of 164 DEGs from the four cohort profile datasets (GSE29001, GSE20347, GSE100942, and GSE38129). DEGs were screened out by GEO2R (https://www.ncbi.nlm.nih.gov/geo/geo2r/) tool, statistically significant DEGs were defined with p < 0.05 and |logFC| > 1 as the cut-off criterion. Venn diagram tool (http://bioinfogp.cnb.csic.es/tools/venny/) was used to identify the overlapping DEGs in the four datasets. Different color areas represented different datasets. The cross areas meant the overlapping DEGs

The four mRNA expression profiles (GSE29001 [12], GSE20347 [13], GSE100942 [14], and GSE38129 [15]), including 63 pairs of ESCC tissues and adjacent normal tissues, were included in this study. Using p < 0.05 and |logFC| > 1 as cut-off criterion [7, 17], we extracted 2594, 1295, 833, and 520 DEGs from the expression profile datasets GSE29001, GSE20347, GSE100942, and GSE38129, respectively. By using Venn diagrams to overlap the DEGs of the four profile datasets, a total of 146 overlapping DEGs were identified (Fig. 1; Table 1), including 120 upregulated genes and 26 downregulated genes. Employing FunRich, we constructed a heatmap of the 120 upregulated and 26 downregulated DEGs using data profile GSE20347 as a reference. Additional file 1: Figure S1 showed the differential distribution of the 146 DEGs.

**Table 1:** 146 DEGs were identified from the four cohort profile datasets, including 120 upregulated genes and 26 downregulated genes in the ESCC tissues compared to normal controls

DEGs	Genes name
Upregulated (120)	NCAPH, P3H4, COL3A1, GGH, ADAM12, NUP155, TPX2, CCNB1, SHCBP1, HMGB3, NETO2, MMP3, IGF2BP2, CENPE, ASPM, SOX4, SPAG5, ANP32E, FAP, TBC1D31, CDCA3, GINS1, ACTL6A, ATAD2, KPNA2, COL1A1, POSTN, BIRC5, STIL, UBE2C, KNTC1, FSCN1, FOXM1, CCNB2, PRC1, HSPBAP1, BORA, CDK1, CHEK1, LRP8, FZD2, CEP55, MINPP1, KIF18B, DNMT3B, TOP2A, FEN1, FANCI, RAD54L, CCNE2, NCAPD2, CDC6, SPC25, CST1, MCM2, MCM4 KIF18A, KIF15, APOBEC3B, AURKA, KIF14, HEY1, BUB1B, TIMELESS, DLGAP5, HJURP, RAD51AP1, AURKB, HDGFRP3, CKS1B, TIPIN, RNASEH2A, MKI67, DTL, RAD51, MEST, MARCKSL1, HMMR, COL5A2, KIF4A, NCAPG2, EXO1, RAD54B, CENPI, GMNN, FZD6, KIF2C, KIF20A, CBS, ORC6, SIX1, TYMS, MELK, CDC20, CENPN, CDH11, NDC80, HOXB7, CCNA2, GTSE1, CDKN3, BUB1, NCAPG, COL1A2, SLC16A1, LAPTM4B, MMP1, TRIP13, APOC1, NEK2, STMN1, CENPF, NUSAP1, EPCAM, CKS2, ISG15, ECT2, ITPR3, KIF23, RPL39L
Downregulated (26)	HSPB8, CRYAB, COL14A1, CXCR2, CRIP2, HLF, ADIRF, AHNAK, RBPMS, FAM189A2, SORBS2, EMP1, FMO2, RRAD, EPS8L1 MEIS1, ADH1B, FAM107A, MXD1, GPX3, ABLIM3, EREG, MAFF, SSBP2, ABLIM1, P2RY14

### Enrichment analysis and PPI network

#### Functional enrichment analysis of DEGs

GO and pathways enrichment were analyzed through multiple databases or software, including DAVID [18], KEGG pathway (http://www.genome.jp/kegg; release 89.0) [29], and FunRich software [19] with p < 0.05 as the cut-off criterion.

**Fig. 2:**
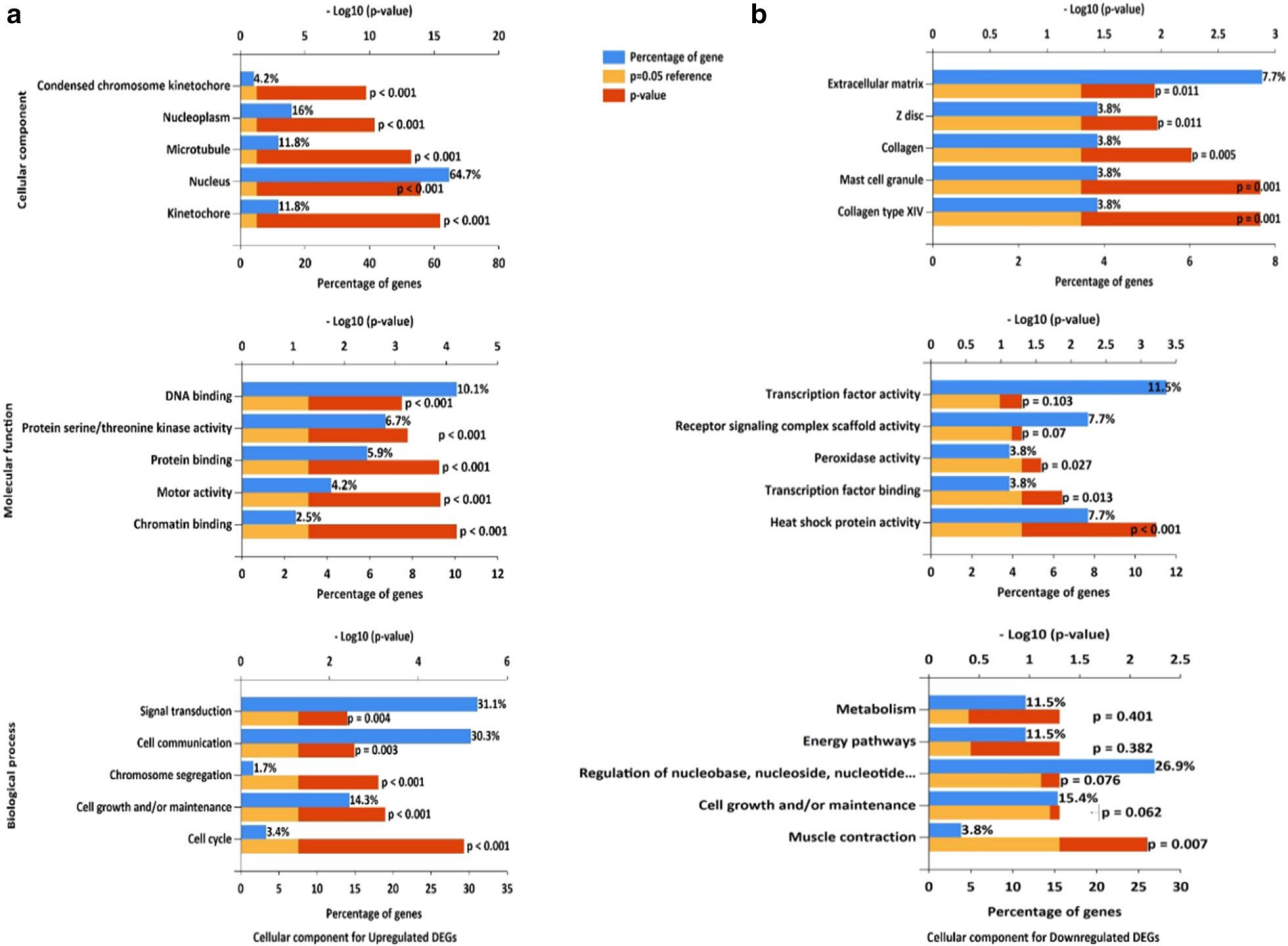
GO analysis and significant enriched GO terms of DEGs in ESCC **a**, upregulated DEGs; **b** downregulated DEGs). GO analysis classified the DEGs into 3 groups (cellular component, molecular function, and biological process)

GO analysis of DEGs classified DEGs into three functional groups: CC, BP, and MF group (Fig. 2). As shown in Fig. 2a, in the CC group, the upregulated genes were enriched in kinetochore, nucleus, microtubule, nucleoplasm, and condensed chromosome kinetochore, whereas the downregulated genes were related to collagen type XIV, mast cell granule, collagen, Z disc, and extracellular matrix (Fig. 2b). In the MF group, the upregulated genes were mainly enriched in chromatin binding, motor activity, protein binding, protein serine/threonine kinase activity, and DNA binding, whereas the downregulated genes were involved in heat shock protein activity, transcription factor binding, and peroxidase activity. As for BP, the upregulated genes were correlated to cell growth and/or maintenance, cell cycle, chromosome segregation, cell communication, and signal transduction. The downregulated genes were significantly connected with muscle contraction. GO term analysis showed that most of the DEGs were enriched in kinetochore, collagen, binding functions, cell cycle, and cell growth. The data were in keeping with the knowledge that abnormality of cell cycle and cell growth regulators was the major cause of tumorigenesis [30]. Moreover, the metabolism of the nuclear components and intercellular substances of cancer cells was different from that in normal cells [31, 32].

**Fig. 3:**
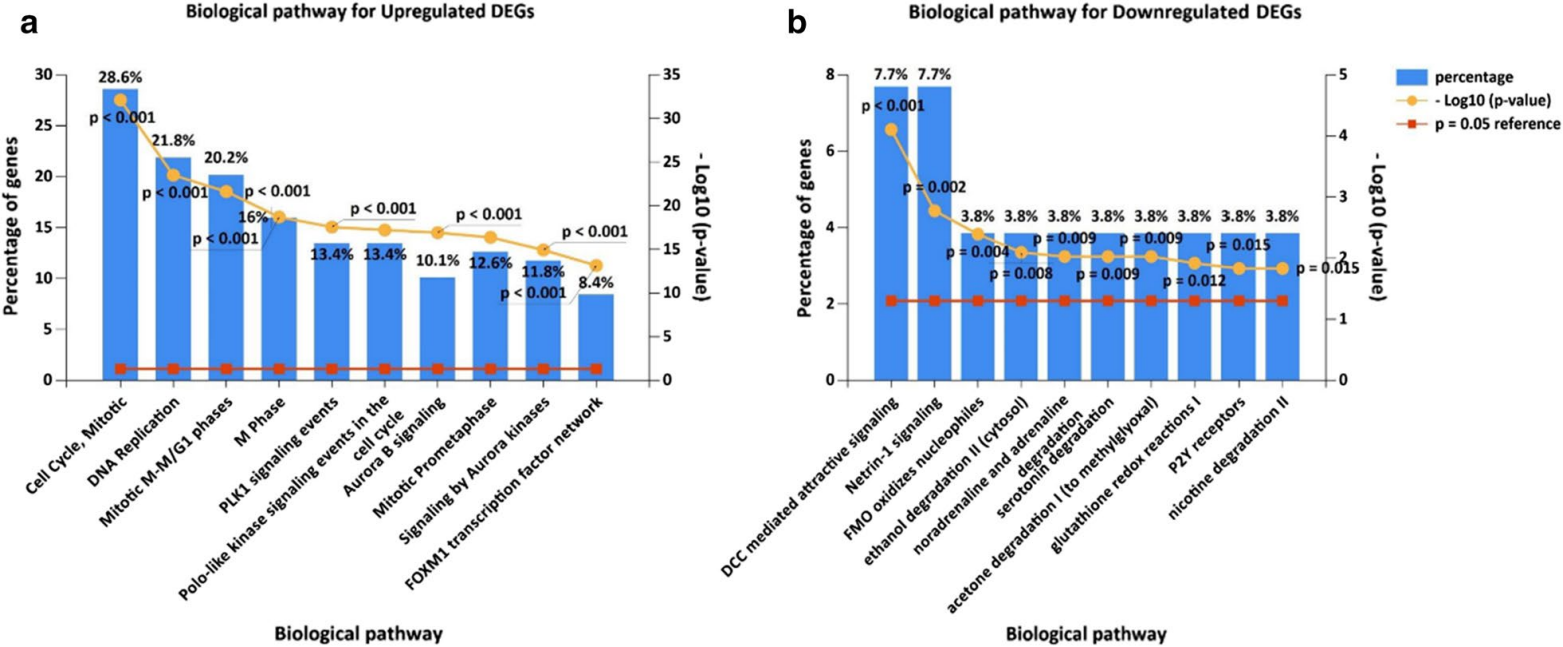
Significantly enriched biological pathway terms of DEGs in ESCC. **a** Biological pathway for upregulated DEGs. **b** Biological pathway for downregulated DEGs. DEGs functional and signaling pathway enrichment were conducted using KEGG pathway (http://www.genome.jp/kegg) and FunRich tool

As for biological pathway enrichment, the upregulated genes were enriched in cell cycle, DNA replication, mitotic M–M/G1 phases, M phase, and Polo-like kinase 1 (PLK1) signaling pathway (Fig. 3a). Previous investigations have demonstrated that some cell cycle-related genes in ESCC development could predict the OS of ESCC patients [33]. Recent evidence also indicated that PLK1 signaling pathway could regulate cell cycle [34]. Moreover, the genes involved in DNA replication system might be useful markers to predict tumor progression [35]. The downregulated genes were enriched in DCC mediated pathway, Netrin-1 signaling pathway, Flavin-containing monooxygenases (FMO) oxidizes nucleophiles, noradrenaline and adrenaline degradation, and ethanol degradation II (cytosol) (Fig. 3b). Previously, we have reported that substance P (SP)/NK-1R signaling could promote the growth and metaseries of ESCC, suggesting that interactions between cancers and nervous system were indispensable for understanding the biological mechanisms of tumorigenesis [36]. Here, we found that the downregulated DEGs were involved in two nervous system-related pathways (DCC mediated signaling pathway and Netrin-1 signaling pathway). Progress toward exploring the links between neuronal activity and oncology might provide new insight into cancer biology. Moreover, specific metabolic activities can be directly involved in cancer progression [35]. The downregulated DEGs were related to metabolic activities such as noradrenaline and adrenaline degradation, suggesting that these metabolism-targeted pathways may be valuable for improving the treatment efficiency.

#### PPI network construction and modules analysis

**Fig. 4:**
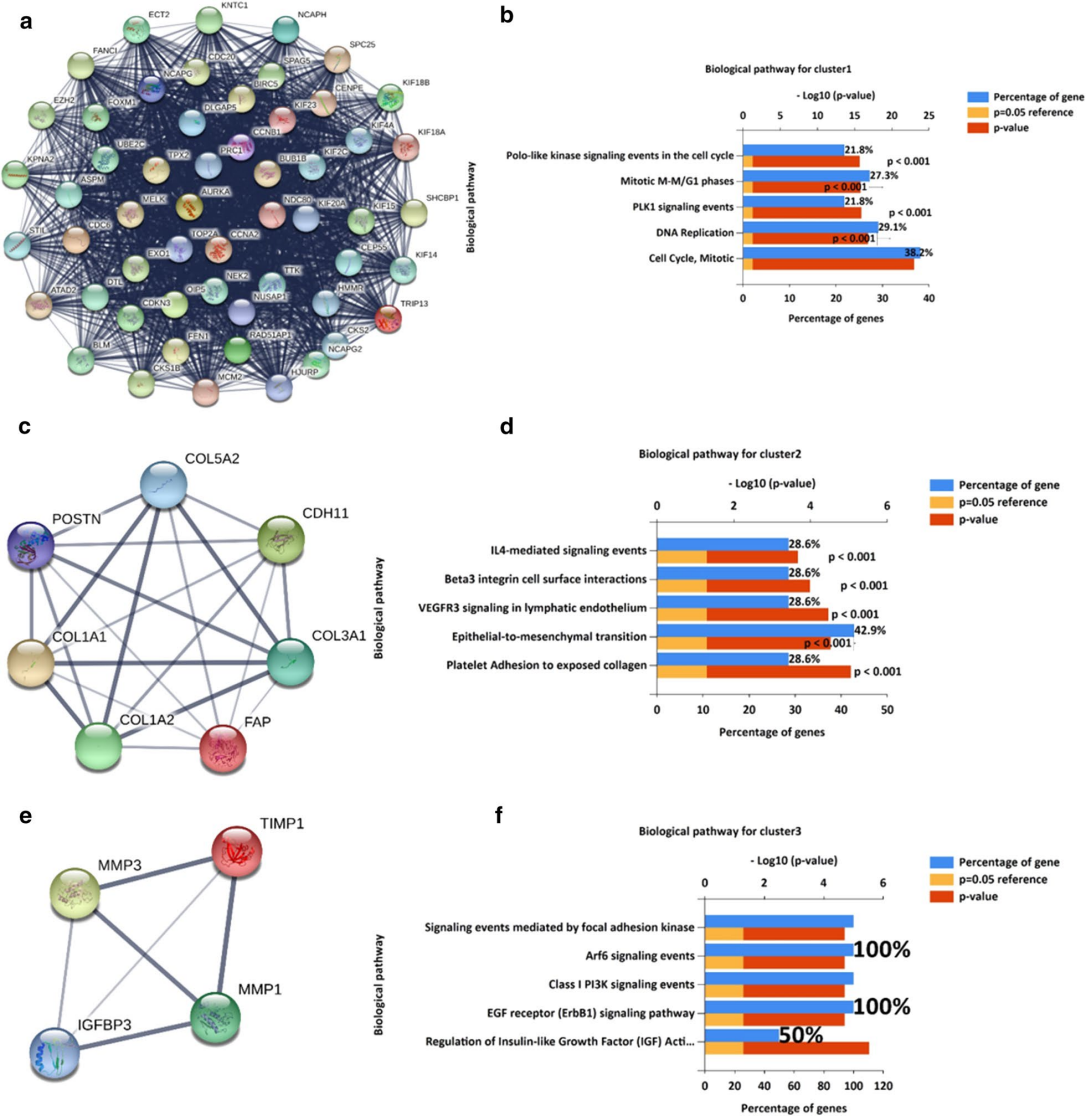
The top 3 modules from the PPI network. **a** Module 1; **b** the top 5 enriched pathways of module 1; **c** module 2; **d** the top 5 enriched pathways of module 2; **e** module 3; **f** the top 5 enriched pathways of module 3

Using STRING database [20] and Cytoscape software [22], totally 146 DEGs were mapped into the PPI network, including 146 nodes and 2392 edges (Additional file 1: Figure S2). The PPI enrichment p-value was 1.0 × 10^−16^. Additional file 1: Figure S3 shows the interaction network of the 146 DEGs and their related genes, allowing us to evaluate their biological functions. For example, AURKA and TPX2 belong to the “Role of Ran in mitotic spindle regulation” pathway, and TPX2 knockdown could inhibit the cell proliferation of ESCC cells [37]. The top three significant clusters within PPI network were selected using MCODE plug-in in Cytoscape software (Module 1, MCODE score = 51.778; Module 2, MCODE score = 7; Module 3, MCODE score = 4). We also analyzed the functions of each module (Fig. 4). Pathway enrichment analysis indicated that Module 1 consisted of 55 nodes and 1398 edges (Fig. 4a, b), which were mainly associated with cell cycle, DNA replication, and PLK1 signaling pathway. Module 2 consisted of 7 nodes and 21 edges (Fig. 4c, d), which were mainly associated with platelet adhesion to exposed collagen, epithelial-to-mesenchymal transition, and VEGFR3 signaling in lymphatic endothelium. Module 3 consisted of 4 nodes and 6 edges (Fig. 4e, f), which were associated with regulation of Insulin-like growth factor (IGF) activity by Insulin-like growth factor binding proteins (IGFBPs), EGF receptor (ErbB1) signaling pathway, and class I Phosphoinositide 3-kinase (PI3K) signaling events.

**Fig. 5:**
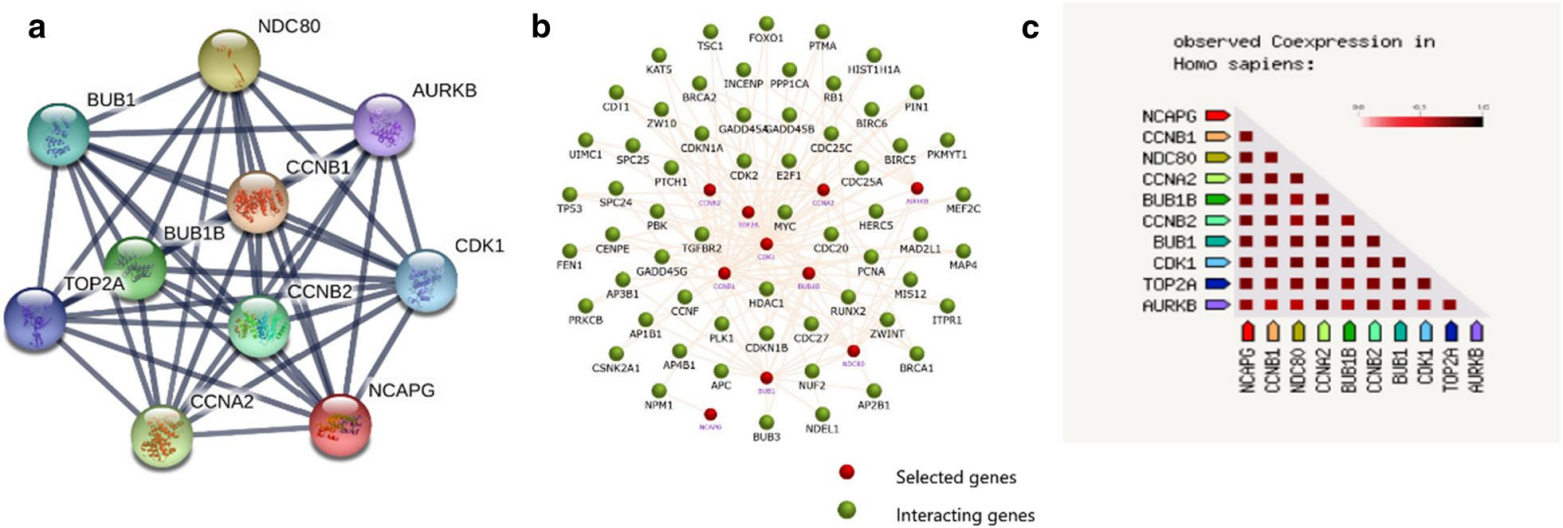
PPI network construction and co-expression analysis for the 10 hub genes in ESCC. **a** A total of 10 hub genes with higher degree of connectivity were selected and filtered into the PPI network complex using the STRING online database. **b** The PPI network of the 10 hub genes and their related genes, created by the FunRich software. **c** The co-expression analysis of 10 hub genes using the STRING online database

Using cytoHubba software, ten genes (Cyclin-dependent kinase 1 (CDK1), Cyclin B1 (CCNB1), DNA topoisomerase II alpha (TOP2A), Cyclin B2 (CCNB2), BUB1 mitotic checkpoint serine/threonine kinase (BUB1), Cyclin A2 (CCNA2), Non-SMC condensin I complex subunit G (NCAPG), Aurora kinase B (AURKB), NDC80, and BUB1B) with higher degree of connectivity were identified as hub genes (Table 2). Moreover, the PPI network of ten hub genes was established using Cytoscape software (Fig. 5a). The interaction network of ten hub genes and their related genes was also established by the FunRich (Fig. 5b). The related genes here were defined as these genes connected to the hub genes. The hub genes and the related genes could be enriched in biological pathways according to the enrichment functions of FunRich tool. The gene co-expression analysis of the ten hub genes performed by STRING database showed that these genes might be actively interacted with each other (Fig. 5c). The above findings suggested that these hub genes might play a crucial role in ESCC progression. For example, AURKB was found to increase in the early development stage of ESCC and might influence the initiation of ESCC [38]. Interestingly, two important genes (BUB1 and BUB1B), which affect the chemotherapy of ESCC [39], could interact with AURKB according to the Fig. 5a. The pathways consisted by these three genes might be important for improving the drug sensitivity of early ESCC.

**Table 2:** Top 10 hub genes with higher degree of connectivity

Genes	Degree	p-value
CDK1	80	< 0.001
CCNB1	78	< 0.001
TOP2A	77	< 0.001
CCNB2	76	< 0.001
BUB1	76	< 0.001
CCNA2	76	< 0.001
NCAPG	75	< 0.001
AURKB	75	< 0.001
NDC80	74	< 0.001
BUB1	74	< 0.001

### Genetic information and hub genes expression

**Fig. 6:**
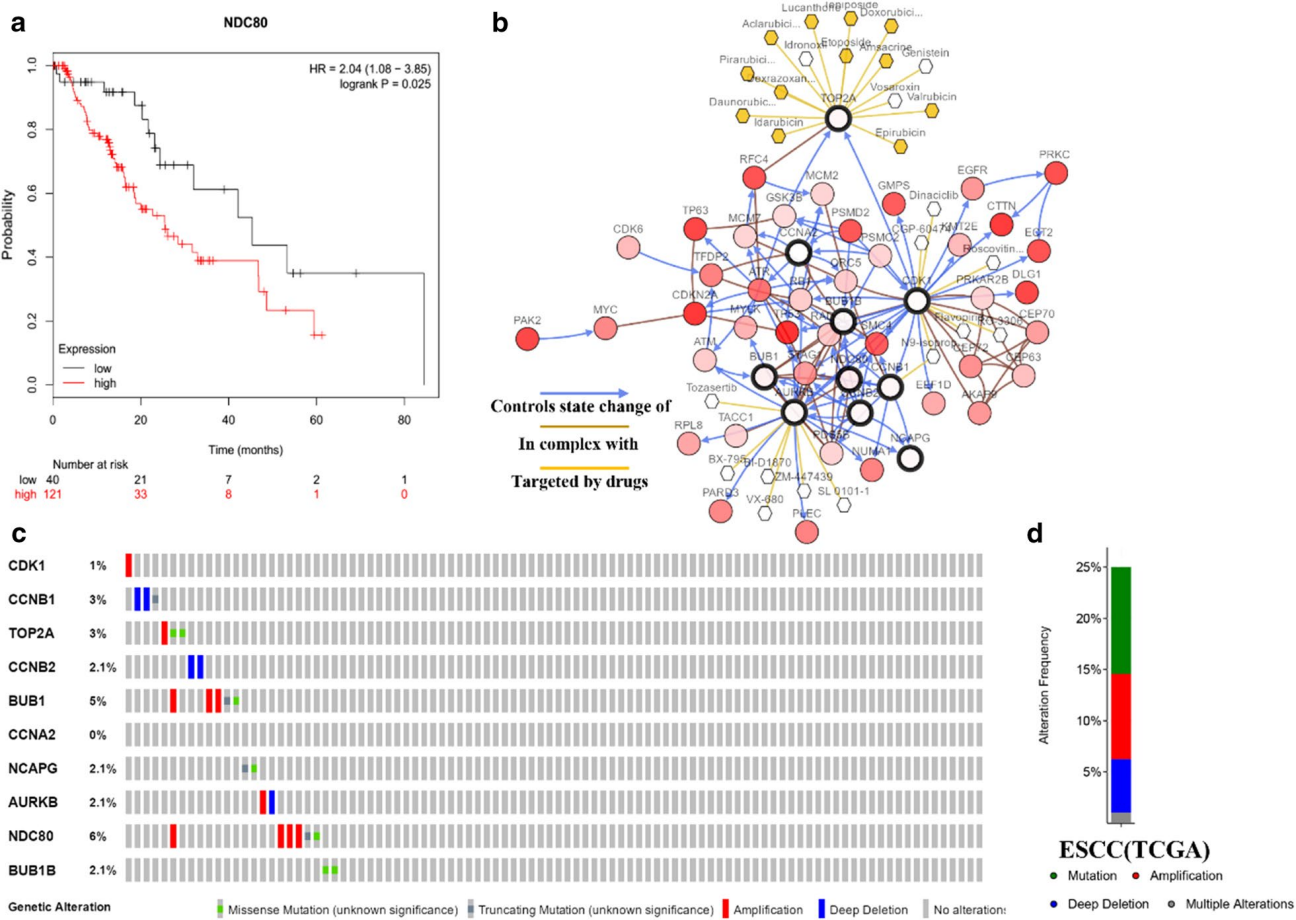
**a** High expression of NDC80 was significantly associated with poor OS in ESCC patients, using a Kaplan–Meier curve and a log-rank test (Kaplan–Meier-plotter website; http://kmplot.com/analysis/). **b** The network contained 50 nodes, including 10 hub genes and the 40 most frequently altered neighbor genes. The relationships between 10 hub genes and drugs were also presented. **c** A visual summary across a set of ESCC (data from esophageal squamous cell carcinoma, TCGA, Provisional) showed the genetic alterations connected with the 10 hub genes which were altered in 24 (25%) of 96 sequenced cases/patients (96 total). **d** An overview of changes in the 10 hub genes in the genomics datasets of ESCC in TCGA database

Kaplan–Meier-plotter website (http://kmplot.com/analysis/) was used to analyze the prognostic information of the ten hub genes. The result showed that the upregulated NDC80 was closely related to the OS of patients with ESCC (Fig. 6a). The deregulation of NDC80 caused by amplification and mutation might lead to poor OS. The remaining 9 genes presented similar trends to that of NDC80, but not statistically significant (Additional file 1: Table S2). Then, we used cBioPortal to enquiry the genetic alterations of the hub genes. And Fig. 6b presented the network constructed by the 10 hub genes and their 40 most frequently altered neighbor genes. Besides, drugs targeting the 10 genes were illustrated. Figure 6b showed that only TOP2A, CDK1, CCNB1, and AURKB were identified as chemotherapy targets currently. We therefore supposed that the other 6 genes (CCNB2, BUB1, BUB1B, CCNA2, NCAPG, and NDC80) might be the novel targets in the future. Figure 6c, d presented the alteration information of the ten genes. The ten hub genes were changed in 24 (25%) of 96 sequenced patients (96 total). NDC80 and BUB1 were changed most often (6% and 5%), these include amplification, mutation and so on.

**Fig. 7:**
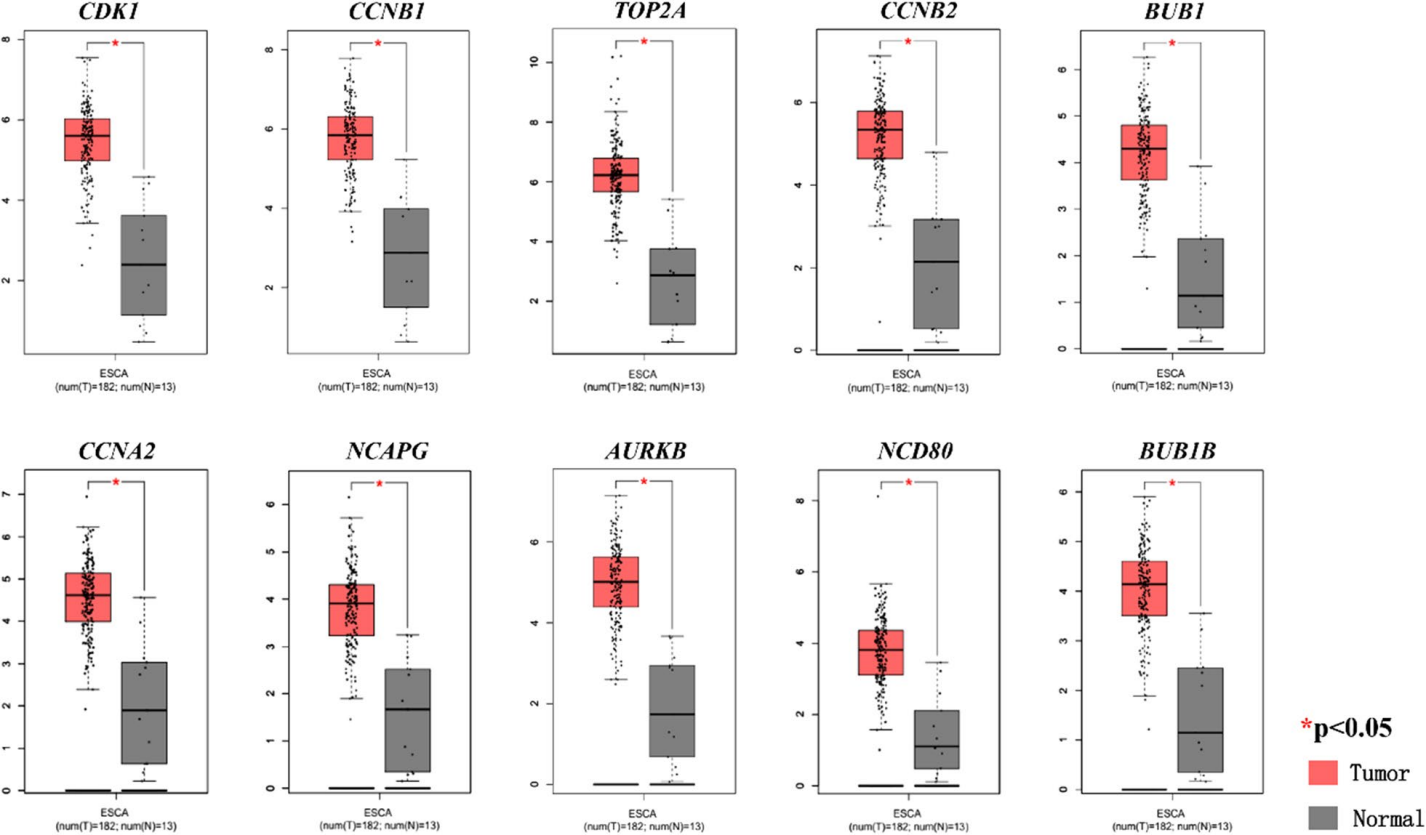
Validation of the hub genes in the Cancer Genome Atlas (TCGA) database. Box plots showed the mRNA expressions of the 10 hub genes using data from the TCGA database in GEPIA2 (http://gepia2.cancer-pku.cn/#index). The validation results of 10 hub genes were in accordance with the profiles in our study, and their p-values < 0.05

We further conducted the expression analysis using the data from GEPIA2 database. The expression levels of the 10 hub genes were significantly different between ESCC and normal tissues (Fig. 7). The expression trends of the 10 genes from GEPIA2 database were in accordance to the data in GEO datasets. Real-Time PCR results revealed that the mRNA expressions of the 9 hub genes (except for CCNA2) were upregulated in EC109 cells (ESCC cell line) as compared to Het-1A cells (esophageal squamous epithelial cell line) (Additional file 1: Figure S5). In addition, the expression levels of the 10 hub genes in ESCC were positively correlated with each other using GEPIA2 database (data not shown).

### miRNA–hub genes network

**Fig. 8:**
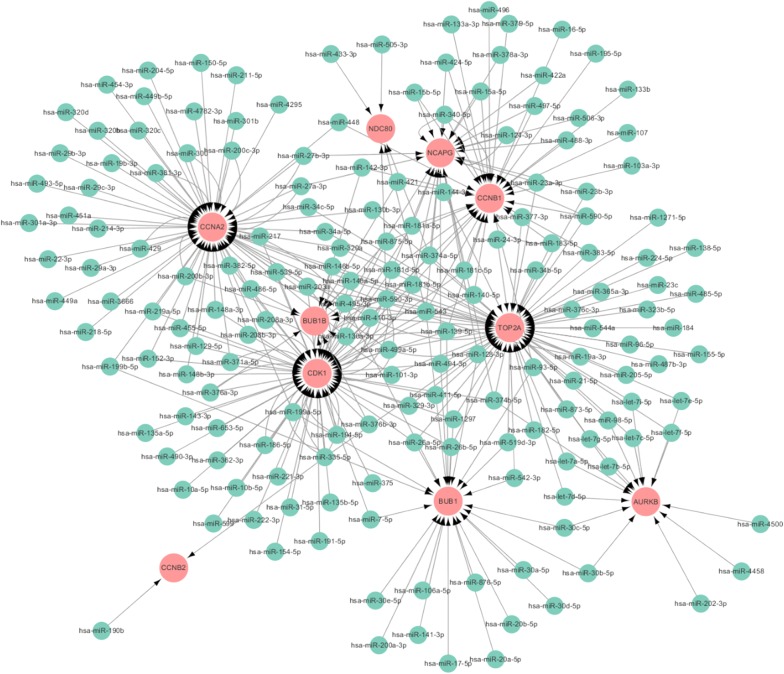
miRNA–hub gene interaction network of ESCC. The green circular node represented the miRNA. The red circular node represented the hub gene. The arrow represented the interaction between the miRNAs and hub genes

To investigate the regulatory relationships of identified hub genes and miRNAs, four miRNA targets prediction databases was used to predicted the targeted miRNAs of hub genes. The miRNAs predicted by at least two databases were selected as the targeted miRNAs of hub genes. The co-expression network based on the correlation analysis between the hub genes and miRNAs was constructed by Cytoscape software (Fig. 8). The numbers of miRNAs and mRNAs in the network were 175 and 10, respectively. In the network, the numbers of arrows in the networks indicated the contribution of one miRNA to the surrounding hub genes, and the higher the degree, the more central the hub gene was within the network. CDK1, TOP2A, and CCNA2 were identified as the three hub genes which were targeted by the most miRNAs. miR-543, miR-495-3p, and miR-590-3p were the top three miRNAs with the most target genes. Previously, Ma et al. demonstrated that miR-219-5p/CCNA2 axis could inhibit the cell proliferation and cell cycle distribution of ESCC cells, highlighting the role of CCNA2 in cell cycle and tumor growth [40]. In addition, miR-543 could facilitate cell mobility and invasion of ESCC by repressing PLA2G4A [41]. Therefore, the miRNA–hub genes network could provide powerful basis for understanding the molecular mechanisms of ESCC.

### Drug-gene interaction

Using the 10 hub genes to explore the drug-gene interactions, 33 drugs for possibly treating ESCC were compiled and selected (Table 3). Promising targets of these drugs include CDK1, TOP2A, CCNA2, and AURKB. Among these four genes, CDK1, TOP2A, and AURKB have been currently considered as the drug targets according to the cBioPortal database (Fig. 6b). The final list comprised only the drugs which were approved by FDA, and several drugs have been tested in clinical trials (teniposide, etoposide, paclitaxel, and epirubicin). Additionally, Table 3 showed that most of the drugs (28/33) might target TOP2A in an inhibitory manner. Among the listed drugs, paclitaxel was considered as a potential drug for cancer therapy based on its interaction with TOP2A [42]. Etoposide, another inhibitor of TOP2A, might inhibit the progress of cancer by inducing DNA damaging [43]. Additionally, TOP2A might be an important therapeutic target in etoposide resistant breast cancer [44]. Using STITCH database, we constructed an extended downstream network of TOP2A to investigate the additional effects caused by TOP2A inhibition. Our model showed that TOP2A inhibition might have possible downstream influence on DNA topoisomerase I (TOP1), DNA topoisomerase II beta (TOP2B), Ubiquitin C (UBC), Proliferating cell nuclear antigen (PCNA), Small ubiquitin-like modifier 1 (SUMO1), and SUMO2 (Additional file 1: Figure S4). According to the network, amsacrine, levofloxacin, dexrazoxane, and etoposide might act as key regulators in these processes. To the best of our knowledge, few TOP2A inhibitors have been tested for ESCC treatments. Some of them were even not to be considered as anti-cancer drugs (such as levofloxacin and dexrazoxane). The data might provide new clues for targeted therapy in ESCC patients.

**Table 3:** Candidate drugs targeting hub genes

Number	Gene	Drug	Interaction types	Approved?	Scores^a^	Ref. (PubMed ID)
1	CDK1	ELTROMBOPAG	Agonist	FDA	1	–
2	CDK1	ROMIPLOSTIM	Agonist	FDA	1	–
3	TOP2A	DOXORUBICIN HYDROCHLORIDE	Inhibitor	FDA	13	–
4	TOP2A	TENIPOSIDE	Inhibitor	FDA	12	8702194; 16271071; 17361331; 17514873; 11752352; 16480143; 9426516
5	TOP2A	ETOPOSIDE	Inhibitor	FDA	12	8823806; 9485461; 8870683; 9494516; 9426516
6	TOP2A	VINCRISTINE	–	FDA	10	9494516
7	TOP2A	DOXORUBICIN	Inhibitor	FDA	9	–
8	TOP2A	NORFLOXACIN	Inhibitor	FDA	6	11752352
9	TOP2A	VALRUBICIN	Inhibitor	FDA	6	11752352; 16019763
10	TOP2A	LEVOFLOXACIN	Inhibitor	FDA	4	11752352
11	TOP2A	ENOXACIN	Inhibitor	FDA	4	18471102; 11752352; 10089819
12	TOP2A	ETOPOSIDE PHOSPHATE	–	FDA	3	–
13	TOP2A	PACLITAXEL	–	FDA	2	–
14	TOP2A	DAUNORUBICIN	Inhibitor	FDA	2	9494516
15	TOP2A	OFLOXACIN	Inhibitor	FDA	2	2847647
16	TOP2A	IDARUBICIN HYDROCHLORIDE	Inhibitor	FDA	2	–
17	TOP2A	PEFLOXACIN	Inhibitor	FDA	2	11752352
18	TOP2A	DAUNORUBICIN HYDROCHLORIDE	Inhibitor	FDA	2	–
19	TOP2A	MITOXANTRONE DIHYDROCHLORIDE	Inhibitor	FDA	2	–
20	TOP2A	AMSACRINE	Inhibitor	FDA	2	1322791; 8823806; 10691026; 8519659; 8632768; 11006484; 11716434; 11752352; 11473732; 1311390
21	TOP2A	PODOFILOX	Inhibitor	FDA	2	16061385; 1334447; 10783066; 11752352; 1845848; 1331331
22	TOP2A	DEXRAZOXANE	–	FDA	2	12911317
23	TOP2A	MITOXANTRONE	Inhibitor	FDA	2	10451375; 11004693; 18687447; 11752352; 9631585; 9494516; 11278845; 9426516
24	TOP2A	LOMEFLOXACIN	Inhibitor	FDA	1	11752352
25	TOP2A	EPIRUBICIN	Inhibitor	FDA	1	14728934; 16234514; 17639997
26	TOP2A	DACTINOMYCIN	–	FDA	1	9494516
27	TOP2A	DAUNORUBICIN CITRATE	Inhibitor	FDA	1	–
28	TOP2A	FINAFLOXACIN	Inhibitor	FDA	1	25808831
29	TOP2A	IDARUBICIN	–	FDA	1	–
30	TOP2A	HYDROQUINONE	–	FDA	1	15833037
31	CCNA2	ETHINYL ESTRADIOL	–	FDA	2	9806355
32	AURKB	SUNITINIB	Inhibitor	FDA	1	–
33	AURKB	SUNITINIB MALATE	Inhibitor	FDA	1	–

^a^The score is the combined number of database sources and PubMed references supporting a given interaction

## Discussion

Numerous researches have been performed to explore the mechanisms of ESCC during the past years, but the trends in incidence and mortality of ESCC is still increasing worldwide. Compared to the previous studies that only focused on several genes or a single cohort, this study selected 4 high-quality gene profile datasets from different research teams to integratedly explore the driven-genes and biological pathways in ESCC. Finally, we identified 146 DEGs (120 upregulated and 26 downregulated). Biological pathway enrichment analysis showed that DNA replication, cell cycle, DCC mediated signaling pathway, and Netrin-1 signaling pathway might paly crucial roles in the progression of ESCC. The PPI network was constructed with 146 nodes and 2392 edges. We then selected the top 3 significant modules from the PPI network, and these three modules were mainly related to DNA replication, cell cycle, PLK1 signaling pathway, EMT process, and ErbB1 signaling pathway, etc. According to the degree of connectivity, the top 10 genes in PPI network were considered as hub genes and they were verified in TCGA database. NDC80 was clearly associated with the poor outcome of ESCC patients. miRNA–hub gene network revealed the importance of epigenetic regulation in ESCC. Additionally, small molecular drugs found here provided new insights into the targeted therapies of ESCC.

Driven-genes play crucial roles during carcinogenesis and progression, and they usually serve a distinct biological function as a module. Using integrated bioinformatics analysis, we have identified 3 important modules. The first module (Fig. 4a) included 55 nodes, and its biological pathways (Fig. 4b) were correlated to DNA replication, cell cycle, and PLK1 signaling pathway. The aberrant cell cycle was one of marked features of tumor cells [45]. In this study, the genes related to cell cycle and mitotic regulation, such as CDK1, CCNB1, CCNB2, and NDC80, were apparently altered in patients with ESCC (Fig. 5c). Importantly, high level of NDC80 predicted poor OS in patients with ESCC. The alterations of these genes included amplification, missense, and mutation. We supposed that the above genes might be the driven-genes for ESCC development. Moreover, DNA replication stress could not only cause cell cycle abnormalities, but also accumulate genome alterations [46, 47]. In this study, the function of Module 1 was associated with the DNA replication, indicating that dysregulation of DNA replication might function as a promoter of sustained proliferation and genome instability in ESCC [46]. PLK1 was one of the most extensively studied genes in cell cycle regulation [48], and it was highly expressed in various cancers, especially in gastric cancer [49], lung cancer [50], and pancreatic carcinoma [34]. Recently, researchers have investigated the PLK1-based targeted therapy and found that PLK1 was involved in several pathways of drug resistance [51].

Module 2 (Fig. 4c) mainly consisted of collagen family members COL3A1, COL1A1, COL1A2, and COL5A2. Dysregulated expression of collagen family members was the foundation of cancer invasion and migration [52]. Many studies have demonstrated that the ectopic expression of the above genes could be the cause of cancer development, resulting in genetic mutations, epigenetic alterations, and activation of oncogenic signaling pathways or processes (such as EMT, extracellular matrix (ECM) remodeling, VEGFR3 signaling pathway, and Wnt signaling pathway, etc.) [53–56]. So far, studies have demonstrated the abnormal expression of collagen family members in several cancers [57–59]. However, their crucial role in ESCC was rarely mentioned [21]. In the future, in-depth studies on the roles of collagen family members in ESCC might provide new clues for inhibiting ESCC.

Module 3 (Fig. 4e) were closely related to IGF activity regulation, ErbB1 signaling pathway, and class I PI3K signaling pathway. Previously, Imsumran et al. have found that the expression level of IGF-I receptor (IGF-Ir) and IGF-II were related to the metastasis, invasion depth, and recurrence in patients with ESCC [60], indicating the potential values of using IGF members as the biomarkers for the prediction of recurrence and outcomes of ESCC patients. Moreover, miR-375 could inhibit the proliferation and migration abilities of ESCC cells through regulating the activity and expression of IGF1R [61]. ErbB1 was overexpressed and mutated in several tumors, including breast cancer [62]. The downstream signaling modules of ErbB included the PI3K/Akt signaling pathway, the phospholipase C (PLCγ) pathway and Ras/Raf/MEK/ERK1/2 pathway [63]. Fichter et al. have found that ErbB inhibitors could inhibit cell migration of ESCC cells through distinct signaling pathways (ERK1/2, Akt, STAT3, and RhoA), suggesting the powerful clues for developing ErbB targeted therapies. Since PI3K/Akt pathway also played important roles in ESCC cell growth, invasion, and migration [64, 65], we thus supposed that Module 3 was the cluster that regulated the growth and metastasis of ESCC cells.

In the study, ten genes were recognized as the hub genes, and their expression levels were all verified in the TCGA database. A list of 33 drugs with potential therapeutic efficacy against ESCC were identified. Among the 10 hub genes, the potential gene targets of the drugs are CDK1, TOP2A, CCNA2, and AURKB. In Table 3, most of drugs were TOP2A inhibitors. However, only a few of TOP2A inhibitors have been used for ESCC. More studies and clinical trials were needed to identify and explore the effective drugs for ESCC. Still, the study might push valuable insights into the individualized treatment and targeted therapy in ESCC, and the conventional drug was of potentially new use.

There were still several limitations worth mentioning in this study. First of all, we majorly explored the functions and potential roles of the hub genes without deeply analyzing the other DEGs. In the future, in-depth studies considering this field is required. Secondly, we only used TCGA data and qPCR assay to validate the expression levels of hub genes, and further experimental studies were required to demonstrate the above findings. Finally, the clinical information of ESCC patients were not deeply analyzed due to the inaccessible of data. Despite this, our study provided novel findings for ESCC studies. Compared to the single dataset studies, this study might provide more accurate results by using integrated bioinformatics analysis. Moreover, the therapeutic targets and drugs found in this study are promising and novel for personalized therapy. Additionally, we constructed the miRNA–hub gene network which might reveal the importance of epigenetic regulation in ESCC.

## Conclusions

Using integrated bioinformatics analysis, the study identified commonly changed 146 DEGs in ESCC, which were enriched in DNA replication, cell cycle, DCC mediated pathway, and Netrin-1 signaling pathway. We also identified 10 hub genes, including CDK1, CCNB1, TOP2A, CCNB2, BUB1, CCNA2, NCAPG, AURKB, NDC80, and BUB1B, that might play important roles in ESCC. The 10 hub genes might function as novel markers and/or targets for the early cancer detection, prognostic judgment, and targeted therapy of ESCC. Additionally, a group of drugs was identified, and they could be potentially utilized for treatment of ESCC patients. And this study provided powerful basis for ESCC studies, and in-depth experimental studies were needed.

## Additional file

**Additional file 1. Figure. S1:** Expression heatmap of the 146 DEGs. **Figure S2**: Construction of PPI network for 146 DEGs in ESCC. **Figure S3**: Construction of PPI network for 146 DEGs and their related genes in ESCC. **Figure S4**: Targetable TOP2A subnetwork. **Figure S5**: Relative mRNA expression levels of the 10 hub genes in EC109 cells compare to that in Het-1A. **Table S1**: The 10 hub genes and corresponding primer sets. **Table S2**: Prognostic information of the 10 hub genes in ESCC patients.

## Data Availability

The authors declare that the data supporting the findings of this study are available within the article.

## Abbreviations

EC: esophageal cancer
ESCC: esophageal squamous cell carcinoma
EAC: esophageal adenocarcinoma
DEGs: differentially expressed genes
GO: gene ontology
KEGG: Kyoto Encyclopedia of Genes and Genomes
PPI: protein–protein interaction
STRING: Search Tool for the Retrieval of Interacting Genes
TCGA: The Cancer Genome Atlas
