# Supplementary material for: Identification of hub genes and therapeutic drugs in esophageal squamous cell carcinoma based on integrated bioinformatics strategy
Source: Cancer Cell Int. 2019 May 22;19:142. doi: 10.1186/s12935-019-0854-6 (PMC6530124; doi:10.1186/s12935-019-0854-6)
Supplement: Supplementary file 1 — Additional file 1. Figure. S1: Expression heatmap of the 146 DEGs. Figure S2: Construction of PPI network for 146 DEGs in ESCC. Figure S3: Construction of PPI network for 146 DEGs and their related genes in ESCC. Figure S4: Targetable TOP2A subnetwork. Figure S5: Relative mRNA expression levels of the 10 hub genes in EC109 cells compare to that in Het-1A. Table S1: The 10 hub genes and corresponding primer sets. Table S2: Prognostic information of the 10 hub genes in ESCC patients. [file 12935_2019_854_MOESM1_ESM.docx]

**Additional file**

**Identification of hub genes and therapeutic drugs in esophageal squamous cell carcinoma based on integrated bioinformatics strategy**

Wanli Yang^1#^, Xinhui Zhao^1#^, Yu Han^2#^*, Lili Duan^1#^, Xin Lu^3^, Xiaoqian Wang^1^, Yujie Zhang^1^, Wei Zhou^1^, Jinqiang Liu^1^, Hongwei Zhang^1^, Qingchuan Zhao^1^, Liu Hong^1^*, Daiming Fan^1^

**Additional file 1: Figure. S1 Expression heatmap of the 146 DEGs.**

**Additional file 1: Figure. S2 Construction of PPI network for 146 DEGs in ESCC.**

**Additional file 1: Figure. S3 Construction of PPI network for 146 DEGs and their related genes in ESCC.**

**Additional file 1: Figure. S4 Targetable TOP2A subnetwork.**

**Additional file 1: Figure. S5 Relative mRNA expression levels of the 10 hub genes in EC109 cells compare to that in Het-1A.**

**Additional file 1: Table S1 The 10 hub genes and corresponding primer sets.**

**Additional file 1: Table S2 Prognostic information of the 10 hub genes in ESCC patients**


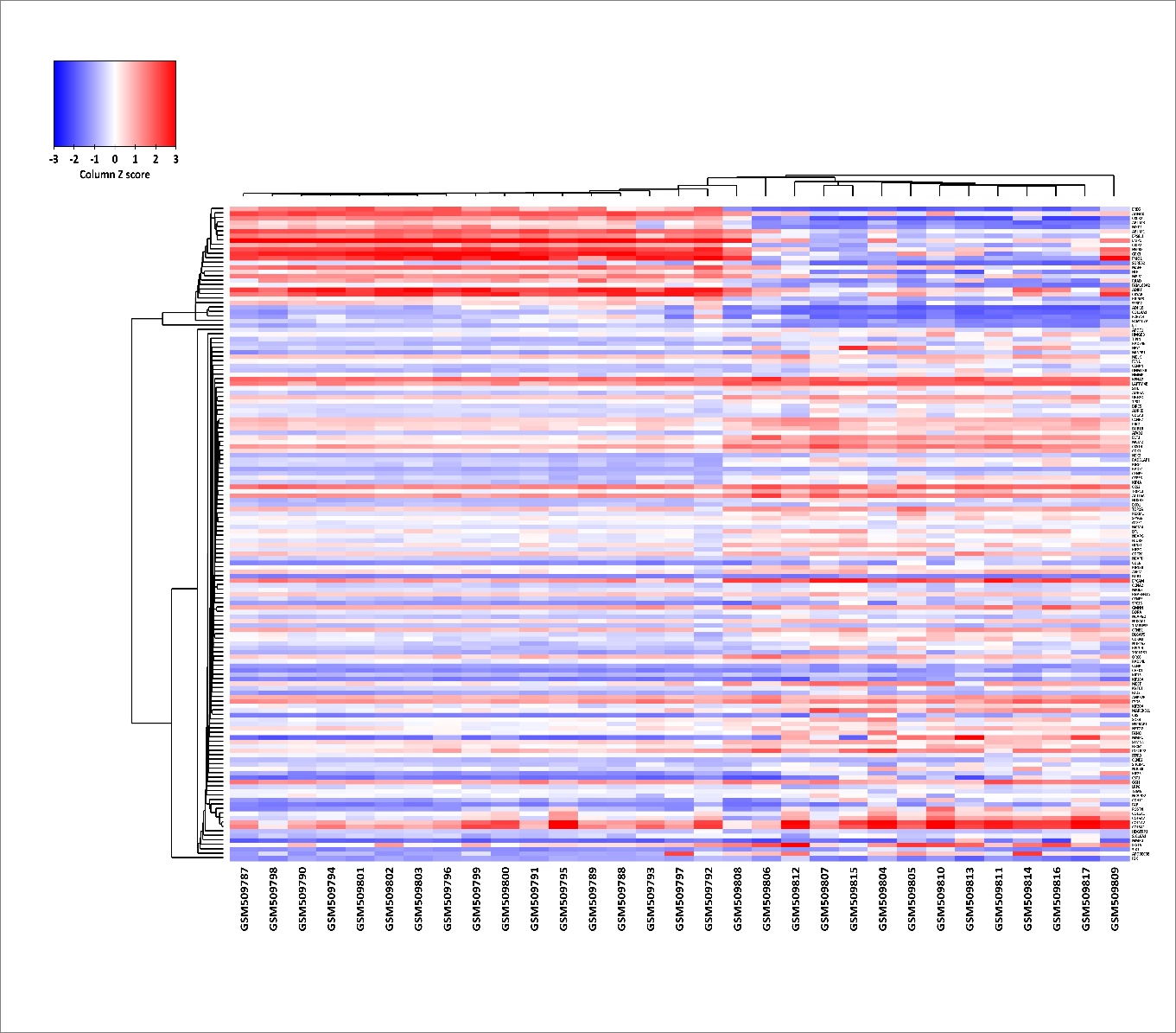


**Figure. S1 Expression heatmap of the 146 DEGs.**


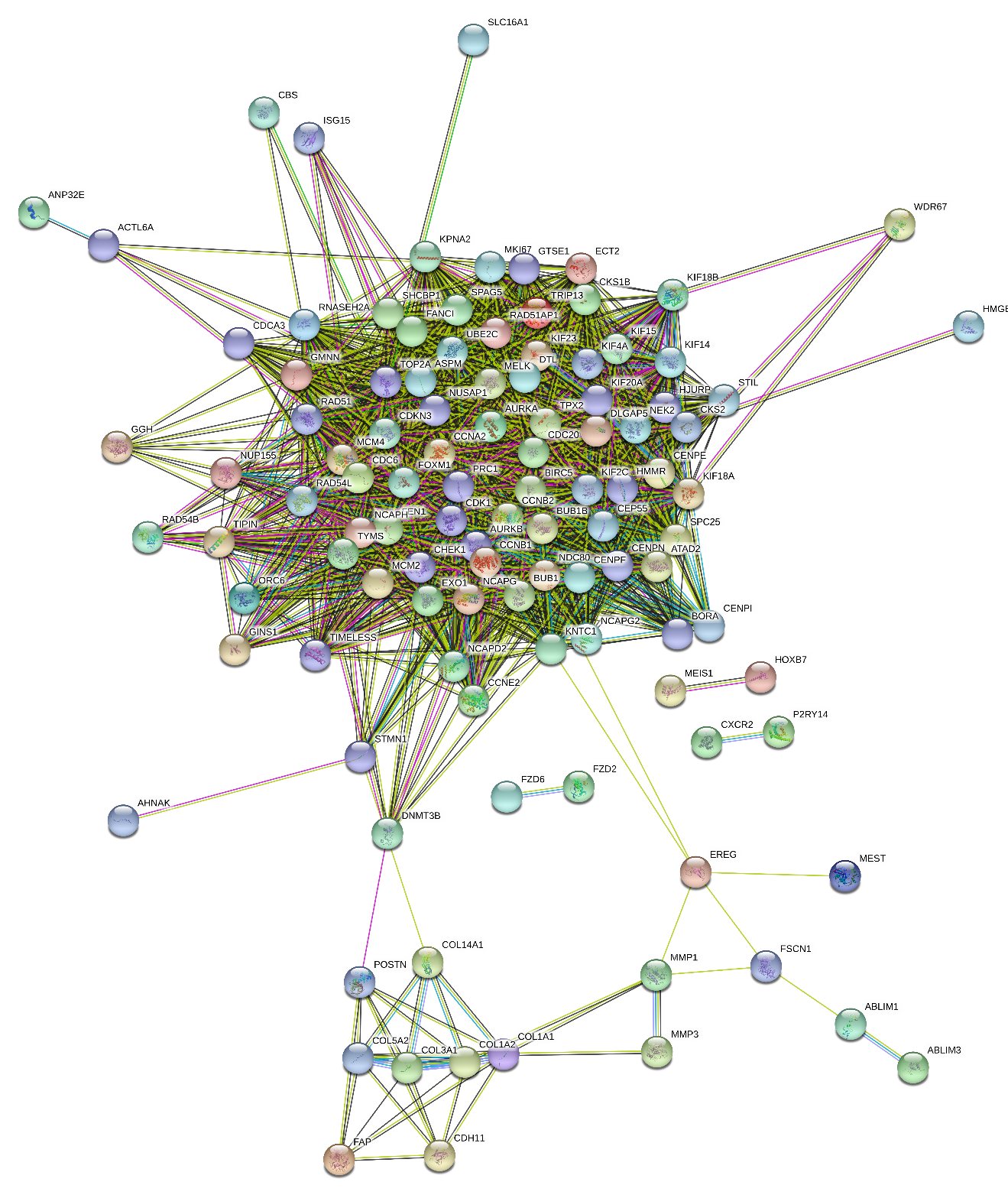


**Figure. S2 Construction of PPI network for 146 DEGs in ESCC.**

A total of 146 DEGs were filtered into the PPI network complex using the STRING online database, including 146 nodes and 2392 edges. The PPI enrichment p-value was 1.0 × 10^-16^.


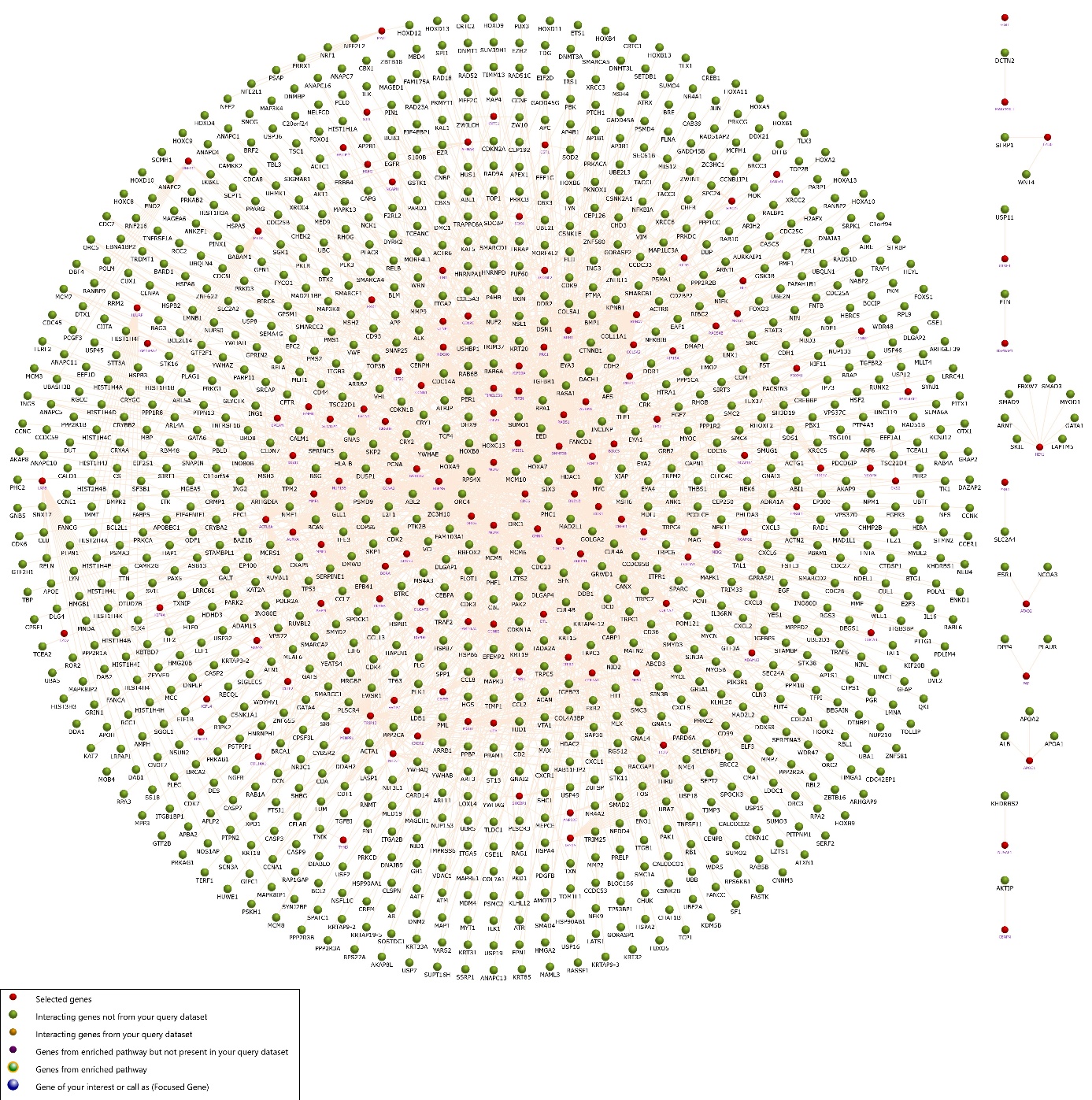


**Figure. S3 Construction of PPI network for 146 DEGs** **and their related genes in ESCC.**

The PPI network of the 146 DEGs and their related genes, created by the FunRich software.

**
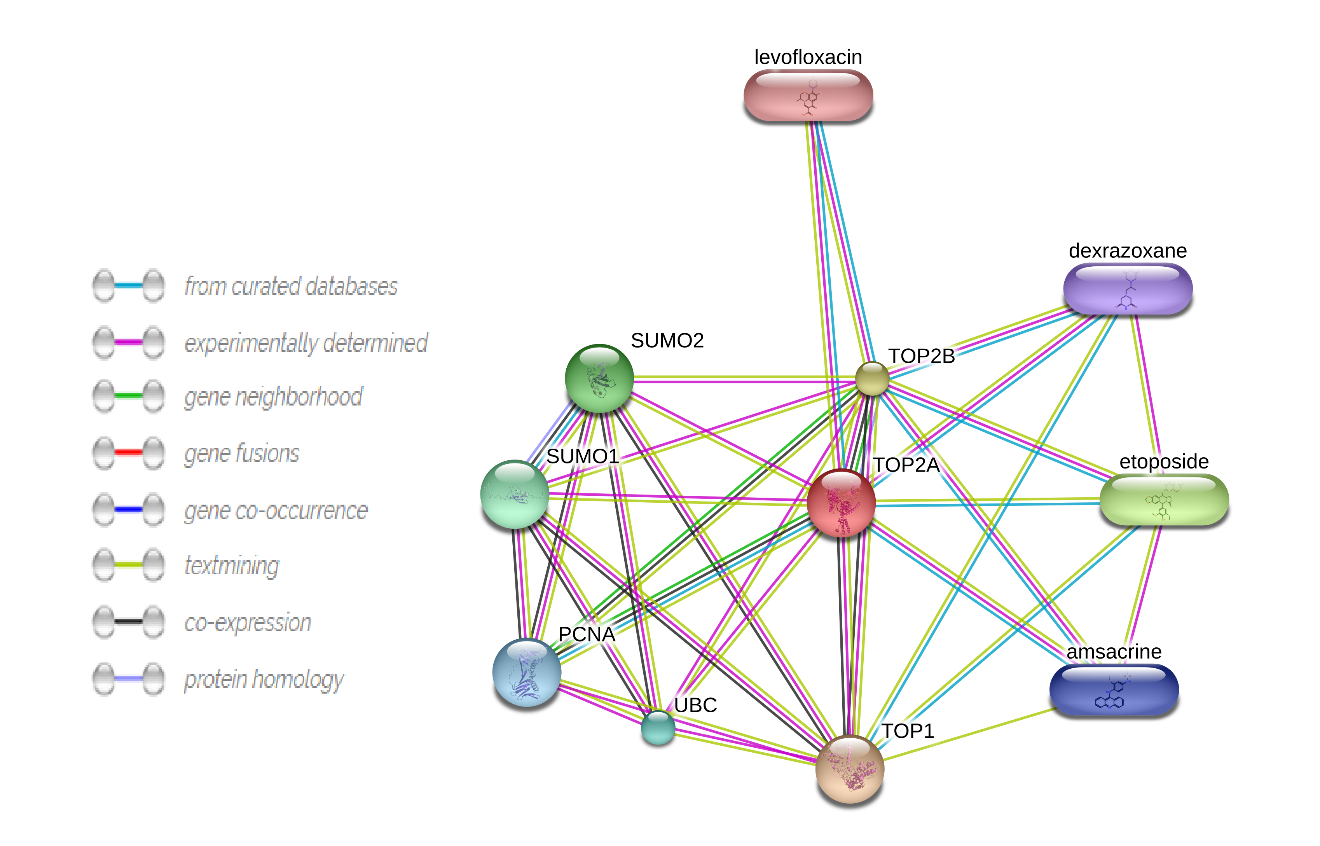
**

**Figure. S4 Targetable TOP2A subnetwork.**

Connecting line color indicates the type of information used to infer the association.


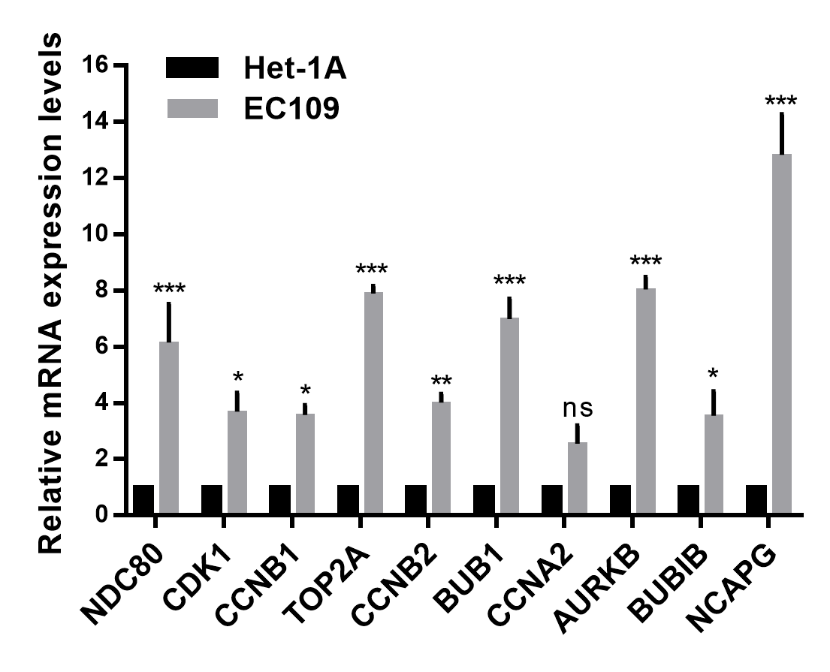


**Figure. S5 Relative mRNA expression levels of the 10 hub genes in EC109 cells compare to that in Het-1A.**

9 hub genes (except for CCNA2) were upregulated in EC109 cells (ESCC cell line) as compared to Het-1A cells (esophageal squamous epithelial cell line). * meant p<0.05; ** meant p<0.01; *** meant p<0.001.

**Table S1 The 10 hub genes and corresponding primer sets.**

| **Genes** | **Primer sets** |
| --- | --- |
| CDK1 | Forward Primer: GGATGTGCTTATGCAGGATTCC |
|  | Reverse Primer: CATGTACTGACCAGGAGGGATAG |
| CCNB1 | Forward Primer: AATAAGGCGAAGATCAACATGGC |
|  | Reverse Primer: TTTGTTACCAATGTCCCCAAGAG |
| TOP2A | Forward Primer: TGGCTGTGGTATTGTAGAAAGC |
|  | Reverse Primer: TTGGCATCATCGAGTTTGGGA |
| CCNB2 | Forward Primer: TGCTCTGCAAAATCGAGGACA |
|  | Reverse Primer: GCCAATCCACTAGGATGGCA |
| BUB1 | Forward Primer: AGCCCAGACAGTAACAGACTC |
|  | Reverse Primer: GTTGGCAACCTTATGTGTTTCAC |
| CCNA2 | Forward Primer: CGCTGGCGGTACTGAAGTC |
|  | Reverse Primer: GAGGAACGGTGACATGCTCAT |
| NCAPG | Forward Primer: GAGGCTGCTGTCGATTAAGGA |
|  | Reverse Primer: AACTGTCTTATCATCCATCGTGC |
| AURKB | Forward Primer: CGCAGAGAGATCGAAATCCAG |
|  | Reverse Primer: AGATCCTCCTCCGGTCATAAAA |
| NDC80 | Forward Primer: TCAAGGACCCGAGACCACTTA |
|  | Reverse Primer: GGGAGCTTGTAGAGATTTCATGG |
| BUB1B | Forward Primer: GCACCGACAATTCCAAGCTC |
|  | Reverse Primer: TGTGCTTCGTTGTGGTACAGA |

**Table S2 Prognostic information of the 10 hub genes in ESCC patients**

| **Genes** | **HR** | **95%CI** | **P value** | **Favor/Un-favor to patients** |
| --- | --- | --- | --- | --- |
| NDC80 | 2.04 | 1.08-3.85 | 0.025* | Un-favor |
| CDK1 | 1.63 | 0.97-2.94 | 0.062 | --- |
| CCNB1 | 1.37 | 0.82-2.3 | 0.23 | --- |
| TOP2A | 0.78 | 0.44-1.4 | 0.41 | --- |
| CCNB2 | 1.2 | 0.72-2.0 | 0.42 | --- |
| BUB1 | 0.75 | 0.44-1.27 | 0.28 | --- |
| CCNA2 | 0.79 | 0.46-1.34 | 0.38 | --- |
| AURKB | 1.45 | 0.85-2.45 | 0.17 | --- |
| BUBIB | 0.75 | 0.45-1.21 | 0.23 | --- |
| NCAPG | 0.61 | 0.37-1.01 | 0.053 | --- |

* Statistically significant
